# Research progress on the use of the optical coherence tomography system for the diagnosis and treatment of central nervous system tumors

**DOI:** 10.1002/ibra.12184

**Published:** 2024-11-22

**Authors:** Jiuhong Li, Ziba Ayi, Gonggong Lu, Haibo Rao, Feilong Yang, Jing Li, Jiachen Sun, Junlin Lu, Xulin Hu, Si Zhang, Xuhui Hui

**Affiliations:** ^1^ Department of Neurosurgery/Department of Cardiovascular Surgery West China Hospital of Sichuan University Chengdu China; ^2^ West China School of Medicine Sichuan University Chengdu China; ^3^ School of Optoelectronic Science and Engineering University of Electronic Science and Technology of China Chengdu China; ^4^ Chengdu Incrpeak Optoelectronics Technology Co., Ltd. Chengdu China; ^5^ Clinical Medical College & Affiliated Hospital of Chengdu University Chengdu University Chengdu China

**Keywords:** animal models, intraoperative real‐time detection, optical coherence tomography system, tumor boundary detection

## Abstract

In central nervous system (CNS) surgery, the accurate identification of tumor boundaries, achieving complete resection of the tumor, and safeguarding healthy brain tissue remain paramount challenges. Despite the expertise of neurosurgeons, the infiltrative nature of the tumors into the surrounding brain tissue often hampers intraoperative differentiation between tumorous and non‐tumorous tissue, thus hindering total tumor removal. Optical coherence tomography (OCT), with its unique advantages of high‐resolution imaging, efficient image acquisition, real‐time intraoperative detection, and radiation‐free and noninvasive properties, offers accurate diagnostic capabilities and invaluable intraoperative guidance for minimally invasive CNS tumor diagnosis and treatment. Various OCT systems have been employed in neurological tumor research, including polarization‐sensitive OCT systems, orthogonal polarization OCT systems, Doppler OCT systems, and OCT angiography systems. In addition, OCT‐based diagnostic and therapeutic techniques have been explored for the surgical resection of CNS tumors. This review aims to compile and evaluate the research progress surrounding the principles of OCT systems and their applications in CNS tumors, providing insights into potential future research avenues and clinical applications.

## Graphical abstract

With the advantages of high‐resolution imaging, efficient image acquisition, intraoperative real‐time detection, and radiation‐free and noninvasive characteristics, optical coherence tomography (OCT) provides accurate diagnosis and effective intraoperative guidance for the minimally invasive diagnosis and treatment of central nervous system (CNS) tumors. These methods include swept‐source OCT, polarization‐sensitive OCT, orthogonal polarization OCT, Doppler OCT, and OCT angiography. This review summarizes the relevant literature and the research progress on the roles and applications of OCT in CNS tumors, provides a synopsis of the advantages of the OCT system in the surgical guidance and diagnosis of CNS tumors based on the detection of the OCT system, and introduces relevant clinical studies on the integration of the OCT system with other neurosurgical therapeutic techniques for the treatment of CNS diseases to provide prospects for future research directions.

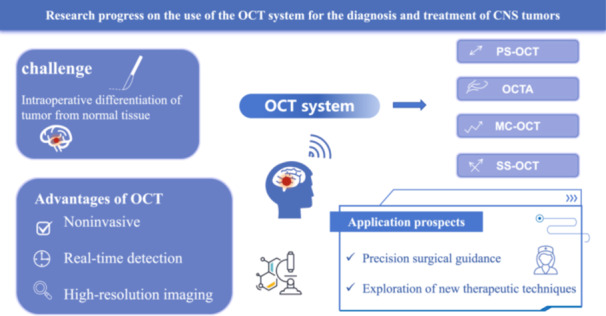

## 1 INTRODUCTION

Currently, the secure microsurgical management of central nervous system (CNS) diseases, particularly tumors, heavily relies on the surgeon's ability to intraoperatively discern tumor boundaries. Specifically, precise differentiation between tumorous and adjacent normal brain tissue is paramount for maximizing the effect of neurological tumor resection, especially in functionally crucial regions. However, the infiltrative nature of malignant tumors within the brain tissue poses a significant challenge, even for skilled neurosurgeons, as microscopic visual identification alone often fails to precisely distinguish tumor tissue from nontumor tissue, resulting in incomplete tumor resection. According to relevant studies, only 23%–50% of lesions can be completely removed via microscopic visual identification solely.^1^, ^2^, ^3^, ^4^

To address these challenges, the application of more sophisticated diagnostic methods can assist surgeons in achieving safer and more complete tumor removal. For instance, diffusion magnetic resonance imaging (MRI) for fiber bundle offers noninvasive tracking of CNS fibers, particularly in the brain's white matter, while ultrasound and computed tomography (CT) scans aid in identifying tumor margins. Nevertheless, these techniques are limited by their detection range, lengthy acquisition time, and suboptimal resolution, which may contribute to inaccuracies. Intraoperative pathology testing, although considered the gold standard for tumor boundary detection, is invasive, time‐consuming, and limited in scope, potentially causing damage to critical functional areas like the brainstem, motor regions, and language centers, leading to severe neurological deficits.

In recent years, optical coherence tomography (OCT) technology has been considered as a solution to overcome the technical limitations of intraoperative pathology examination. OCT has the advantages of noninvasiveness, high resolution, real‐time display, strong continuity, and portability, offering the potential for intraoperative diagnosis, guidance, and postoperative evaluation.^5^ OCT is capable of observing biological tissues in real time in a noninvasive, label‐free manner for surgical guidance with a resolution of up to 1–15 μm and a depth of up to 2 mm.^6^ Therefore, OCT enables high‐resolution and precise identification of tumor‐ and nontumor tissues, which provides new possibilities for brain tumor detection and neurosurgical guidance during CNS tumor surgery, especially for glioma resection.

The overarching purpose of this review is to comprehensively assess the literature on the fundamental principles and applications of OCT in the study of CNS tumors. Specifically, we summarized the research advancements in this domain, synthesized studies on the utilization of OCT in surgical guidance and diagnosis for CNS tumors, and introduced relevant clinical research on the integration of OCT diagnostics with neurosurgical therapies. In addition, we summarized the application and development prospects of artificial intelligence (AI) in OCT systems, with a focus on its potential to enhance diagnostic accuracy, optimize treatment decisions, and drive medical innovations, so as to offer insights into future research directions and clinical implementations of OCT and AI in the field of CNS tumors.

## 2 PRINCIPLES OF THE OCT SYSTEM

**Figure 1:**
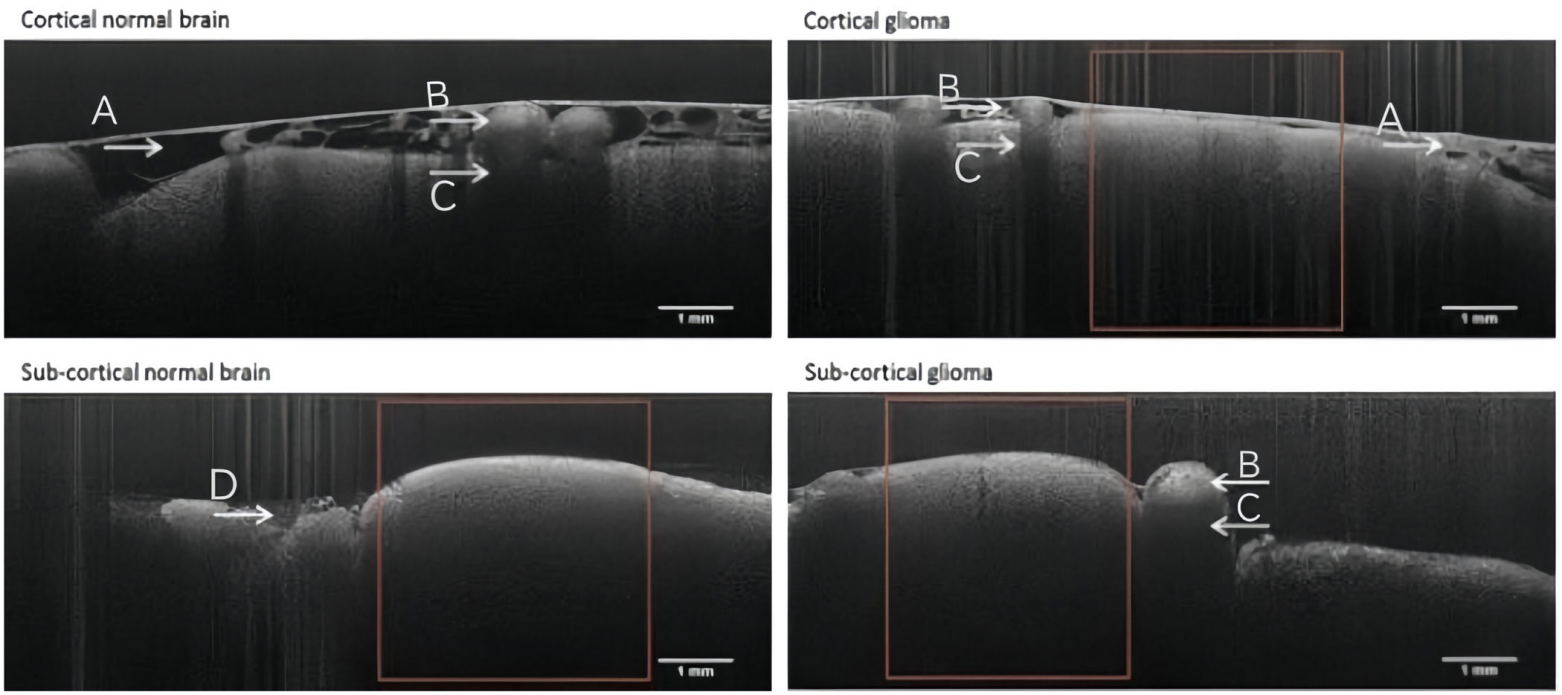
Cortical cross‐sectional optical coherence tomography scans of normal brain tissue and glioma tissue.^8^ The figures on the left show normal tissue, while the figures on the right exhibit glioma tissue. (A) Arachnoid space, (B) blood vessels, (C) vessel shadows, and (D) blood. [Color figure can be viewed at wileyonlinelibrary.com]

For OCT, coherent near‐infrared light is used to detect the tissues in which the light can be reflected, absorbed, and transmitted. The resonance phenomenon occurs between light of identical frequencies. Coherent near‐infrared light is absorbed by hydrogen‐containing groups such as ‐NH, ‐OH, and ‐CH at the vibrational orbital and combined frequency, and the absorption shows summed vibration of the molecule with vibrational absorption. Different tissues (tumors and normal tissues) with varying compositions and percentages of molecular groups, will display differences in the intensity of absorption under infrared spectral irradiation. Consequently, their infrared reflectance spectra will also differ,^7^ making it possible to use the spectra provided by the OCT system to infer the type of tissue being examined (Figure 1). Taking the principle of glioma boundary detection as an example, normal brain tissue around glioma tumors contains more complete myelin sheaths, which are composed mainly of proteins and lipids (including phospholipids, glycolipids, cholesterol, etc.) and have a lower water content,^9^ while tumor areas contain more cells and have greater water content. Additionally, peritumoral infiltrated brain tissue may exhibit edema and destruction of myelin sheaths. Therefore, gliomas, peritumor infiltrated regions, and normal brain tissue differ in molecular composition and the proportions of specific groups, leading to distinct infrared spectral signatures.

It is noteworthy that infrared radiation experiences an exponential decrease in intensity when traversing through tissues, allowing its attenuation coefficient to differentiate between diverse tissue types or physiological variations within identical tissues.^10^ However, this characteristic also presents limitations on OCT systems. Specifically, the imaging depth achieved by OCT is significantly constrained by the attenuation of light scattering, typically reaching 2 mm in most tissue types.^11^ Given that OCT frequently involves imaging in nontransparent tissues with high scattering properties, the imaging depth is primarily influenced by scattering and sensitivity. To enhance image penetration, longer optical wavelengths can be employed to mitigate scattering, but the corresponding resolution may be reduced. Additionally, while OCT imaging may exhibit minor artifacts in the form of strong and weak noise shadows, its reliance on coherent light ensures robustness against variations in ambient light intensity. Consequently, noise spots should initially be manually identified and subsequently addressed through AI.

## 3 APPLICATION OF OCT SYSTEMS AND DERIVED SYSTEMS

OCT systems applied to the study of neurological diseases mainly include swept‐source OCT systems (SS‐OCT), polarization‐sensitive OCT systems (PS‐OCT), orthogonal polarization OCT systems, Doppler OCT systems, and OCT angiography systems.

**Figure 2:**
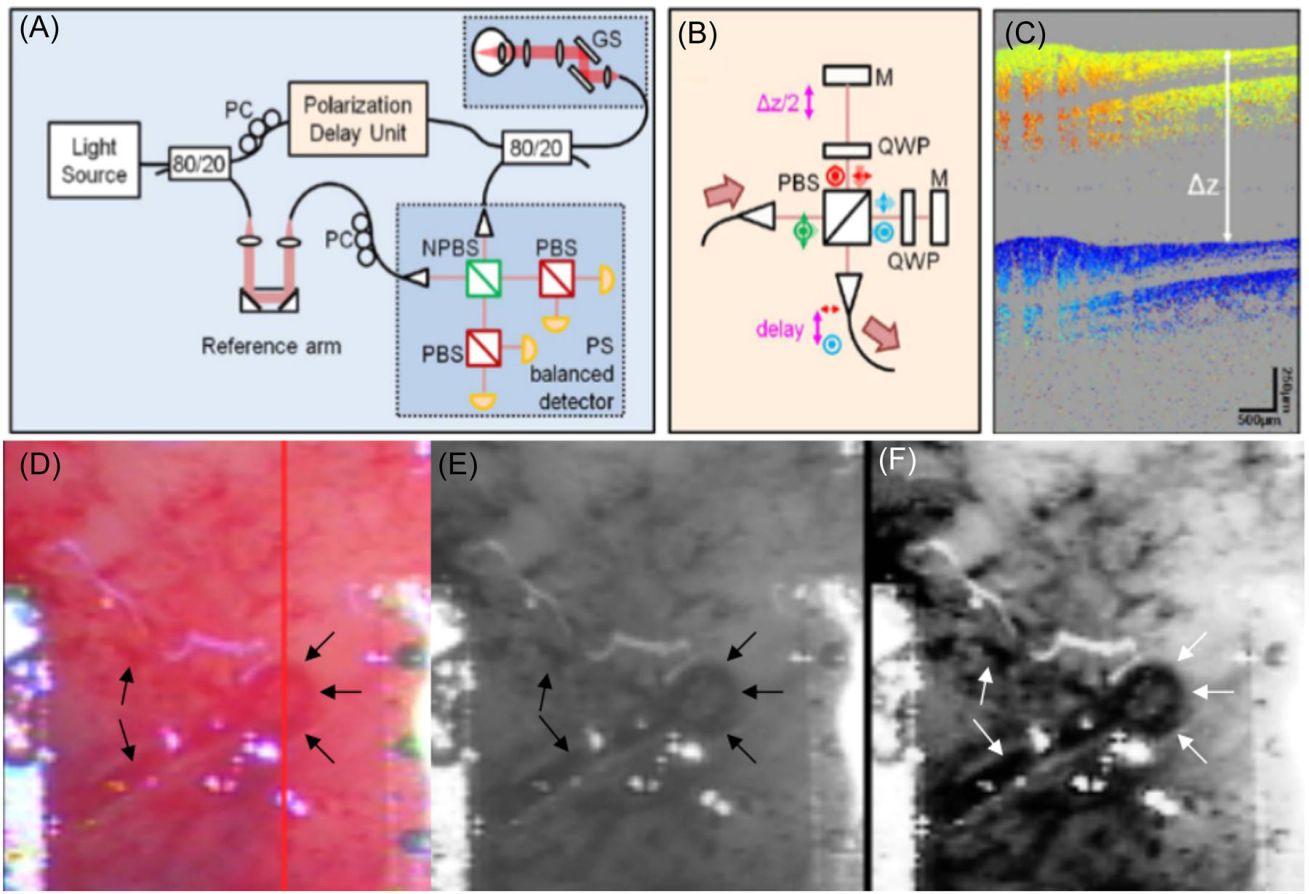
SS‐OCT/FD‐OCT image of a glioma specimen with a passive polarization delay unit (A–C)^12^ and OCT surface map of the glioma specimen showing the vasculature (D–F). (A) Setup program of SS‐OCT. The sample illumination path contains a polarization delay unit. The sample and reference beams interfere at the unpolarized beam splitter and are then split into orthogonally polarized components by the polarizing beam splitter. (B) Passive delay cell scheme. (C) Demonstration of the phase difference between orthogonal detection channels. (D) A general view of the surface of an ex vivo glioma specimen (approximately 5 × 5 mm in size) showing vasculature (arrows). (E) The OCT grayscale sequence surface map shows iso‐ and hyperintensity in the tumor area and hypointensity in the vascular area (arrows). (F) A surface scan enhancement image showed that the tumor area was hyperintensity, and the blood vessel area was hypodense (arrows). OCT, optical coherence tomography; SS‐OCT, Swept‐source optical coherence tomography; SS‐OCT/FD‐OCT, Swept‐source/Fourier domain optical coherence tomography. [Color figure can be viewed at wileyonlinelibrary.com]

Swept‐source/Fourier domain optical coherence tomography (FD‐OCT), also known as optical frequency domain interferometry (OFDI), is usually used for biomedical imaging (Figure 2). The light from the swept source is split equally into two identical beams by the coupler. The two beams of light reflected or scattered from the sample and reference arms form an interference field in the coupler.^13^ The low‐coherence interferometric spectral signals from a broadband swept‐frequency light source are recorded in time using a point detector, and parallel acquisition of longitudinal information within the sample is achieved by the Fourier transform. The imaging speed of this technique is mainly determined by the sweep frequency of the light source.^14^

PS‐OCT can produce images of phase retardance, reflectivity, and optic axis orientation with depth resolution. Moreover, this method detects and identifies polarization‐altered states in tissues and can reveal birefringent properties in samples (mainly related to the reflectivity of light and the anisotropy of the tissue structure), exploiting the optical properties of nerve fiber myelin and allowing quantitative determination of OCT signals.^15^, ^16^ The captured birefringence in biological samples enhances imaging contrast, particularly in tissues with highly coherent microstructures, such as cartilage, teeth, and blood vessel walls. Orthogonal polarization OCT systems are a variant type of PS‐OCT that allows imaging of both the birefringent properties of tissue samples and the altered state of the original polarization produced by cross scattering.^17^, ^18^, ^19^

Doppler OCT systems combine the high‐resolution imaging capability of OCT with the hemodynamic analysis of the Doppler effect, which enables visualization and quantitative measurement of blood flow to provide more comprehensive information for clinical diagnosis. Over 20 years ago, the first phase‐resolved OCT–optical Doppler tomography to image blood flow in blood vessels of human skin was developed with high velocity and sensitivity.^20^ This approach progressed to the development of a phase signal‐based OCT‐angiography (OCTA) technique in 2006, which included methods to minimize sample movement in the axial direction and thus improve image quality.^21^ This technique is used for the quantitative analysis of blood flow in blood vessels and high‐resolution structural reconstruction of vascular networks. Currently, the most notable application of OCT in preclinical cancer research is in the field of angiography.

OCTA imaging relies on the measurement of scattering kinetics, specifically the motion contrast of blood cells, to generate three‐dimensional images of vascular structures, which can offer a method for probing vessel walls in both open surgery and catheter‐based vascular surgery. Due to their characteristics of real‐time detection, high resolution, depth resolution, blood flow determination, and noninvasiveness, these systems have excellent prospects for use in the detection of tumors and cerebral microvessels.^22^ It can also be used to identify cerebral or tumor vessels that differ in morphology, density, and vessel thickness since vessels in tumors are more coiled, with irregular luminal profiles compared to surrounding tissues, aiding in the discrimination between tumor and non‐tumor regions. Geng et al. developed a natural fat nanoemulsion and demonstrated the zero toxicity and human clearance of DSPE‐PEG2000‐COOH‐Intralipid (P‐INT), which was shown to significantly improve OCT imaging contrast and detection resolution, as well as the monitoring of brain diseases and tumor angiogenesis.^23^

The multicontrast optical coherence tomography (MC‐OCT) system, which integrates PS‐OCT with Doppler OCT, can simultaneously resolve depth‐resolved reflectivity, blood flow images, and axial orientation and offer comprehensive 3D optical beam imaging. The micrometer resolution allows visualization of the architecture of the fiber tracts with a high level of detail, which makes it potentially useful in clinical applications. Wang et al.^24^ investigated a dual‐contrast MC‐OCT system based on polarized holding fibers and showed via ex vivo rat experiments that the delay information provided by MC‐OCT provides a more robust distinction between white and gray matter, highlighting the system's potential for improved tissue distinction.

In summary, OCT is a powerful and promising medical imaging technology. It allows for “optical biopsies,” enabling real‐time in situ observation of microstructural and pathological changes in tissues without the need to remove and process the specimen.^25^ Currently, preliminary studies on the application of OCT technology in the diagnosis and treatment of neurological diseases have focused mainly on the following aspects: (1) providing real‐time intraoperative feedback on brain tissue imaging, such as distinguishing between tumor tissue and normal brain tissue, peritumoral infiltrated brain tissue and normal brain tissue, and assessing the degree of white matter damage; (2) providing rapid intraoperative characterization of fresh tissue samples; and (3) assisting in the guidance of stereotactic biopsy and directing surgical interventions.

## 4 PROGRESS OF OCT AND DERIVED TECHNIQUES IN INTRACRANIAL TUMOR BOUNDARY DETECTION

**Figure 3:**
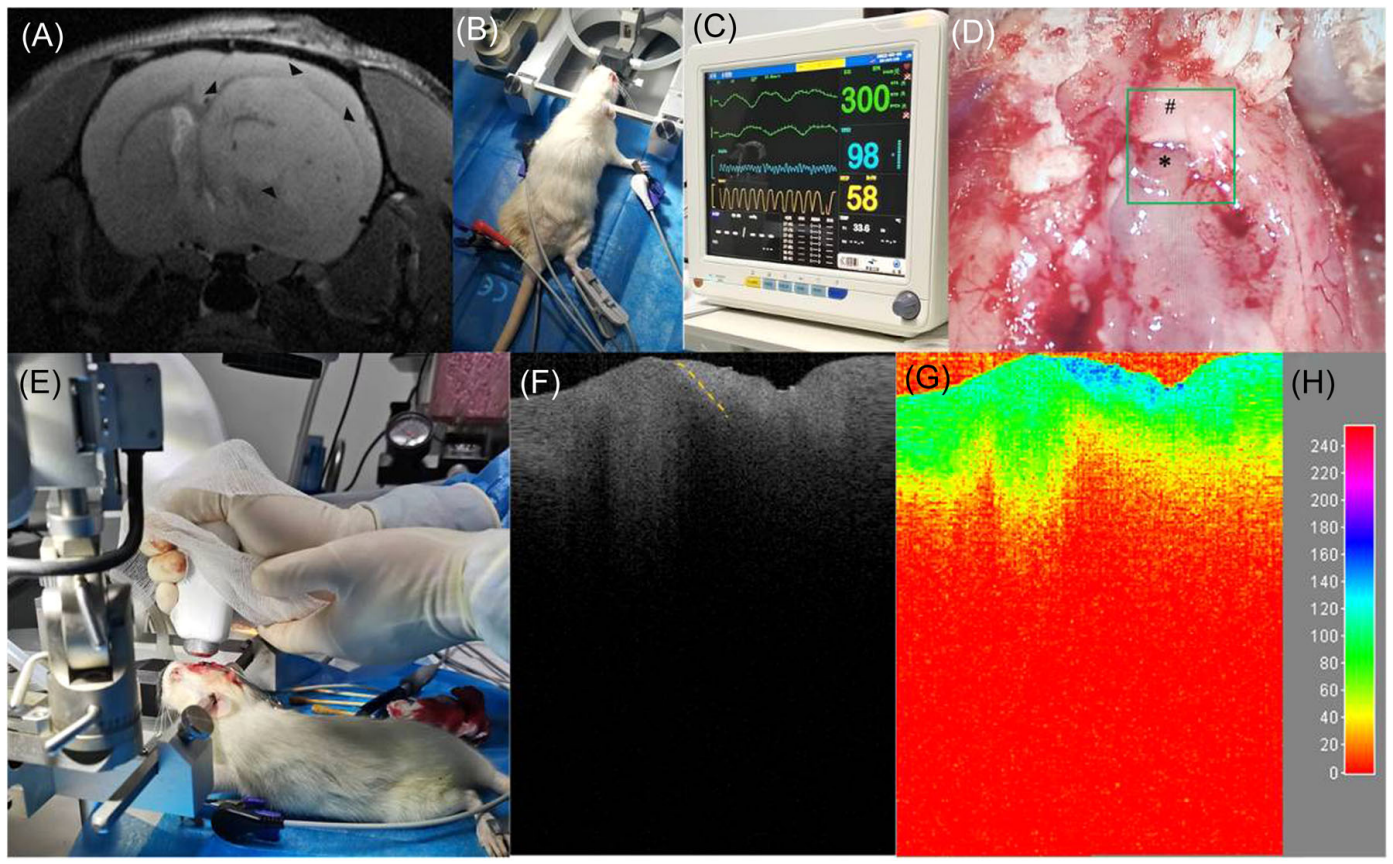
Application of the optical coherence tomography (OCT) system in detecting tumor boundaries in glioma in an SD rat in vivo. (A) Preoperative intracranial magnetic resonance T2 imaging showing a glioma located in the right frontal lobe in an SD rat (triangular arrows). (B) The rat was properly immobilized after induction of anesthesia, and the anesthesia was maintained by a mask. The limbs were affixed with electrocardiograph (ECG) monitoring clips and oxygen saturation detection clips, and an anal temperature monitor was inserted into the anus. (C) The ECG monitor displayed the animal's electrocardiogram, heart rate, oxygen saturation, respiratory rate, and body temperature so that we could adjust anesthesia parameters such as isoflurane concentration and oxygen flow according to the monitoring of vital signs during surgery to ensure safe tumor boundary detection in vivo. (D, E) After craniotomy, the bone window was removed, the brain tissue covering the surface of the tumor was removed, the tumor area was exposed, and the area of interest (green box) was detected in real time without any touching the OCT system. (F) A B‐scan grayscale map of this region shows the boundary between the tumor and the peritumoral normal brain tissue (orange dashed line). The left side of the dashed line is the hypointense region indicating the tumor (* in D), and the right side of the dashed line is the hyperintense region indicating the peritumoral normal brain tissue (# in D). (G) The two‐dimensional color map reconstructed using ImageJ software showed that the tumor area was dominated by lower color levels (e.g., green), while the peritumoral normal brain tissue contained higher color levels (e.g., blue). The (H) chart is a reference scale for the color visualization transformation of the gray value from 0–255 of the (G) chart. ECG, electrocardiography; OCT, optical coherence tomography. [Color figure can be viewed at wileyonlinelibrary.com]

The literature focusing on the application of OCT and derived techniques for intracranial tumor boundary detection is summarized in Table 1. The most widely investigated area of detection by the OCT system is tumor boundary detection in animal models in vivo (Figure 3).

**Table 1:** Summary of current pieces of literature on OCT systems for brain and CNS tumors detection.

References	Type of research	Subject of research	Type of organization	Mode of analysis	Results of research
Bizheva et al.^26^	In vitro (human specimen)	Fibrous meningiomas, 3; atypical meningiomas, 3; gangliogliomas, 2	Tumors, Healthy brain tissue	Qualitative, visualization	Visualization of tumor cells located at different depths within the tissue or larger morphological features, distinguishing between healthy and pathological brain tissue, demonstrates the successful application of OCT as an in vivo optical biopsy tool in neurosurgery.
Bohringer et al.^7^	In vivo (human)	9 patients (Glioma grade 3–4)	Cortex, white matter, tumors	Qualitative, quantitative	Correlation between optical tissue analysis scores and histological results. (*χ* ^2^ test; *r* = 0.99).
Kut et al.^27^	In vivo (animals) In vitro (human specimen)	Living‐ 5 rats. In vitro‐ 32 patients with gliomas (grades 2–4)	Cortex, white matter, tumors	Quantitative, color‐coded images	Sensitivity and specificity 100%, 80% (low‐grade glioma) 92%, 100% (high‐grade glioma)
Yashin et al.^19^	In vitro (human specimen)	30 patients with gliomas (grades 1–4)	Cortex, white matter, tumors	Quantitative, color‐coded images	Sensitivity and specificity: 95.6–90.1%/81.3–87.5% for all tumors 100%/100% for tumor tissue without necrotic areas 91.5–81%/81–87.5% for tumor tissue with necrotic areas
Almasian et al.^8^	In vivo (humans)	5 patients with gliomas (grades 2–4)	Cortex, white matter, tumors	Quantitative	Mean attenuation values were 3.8 ± 1.2 mm^−1^ for normal brain tissue in the cortical layer and 3.6 ± 1.1 mm^−1^ for gliomas. Mean attenuation values were 5.7 ± 2.1 mm^−1^ for normal brain tissue in the subcortical layer and 4.5 ± 1.6mm^−1^ for gliomas.
Juarez‐Chambi et al.^28^	In vitro (human specimen)	21 patients with gliomas (grades 2–4)	Cortex, white matter, tumors	Quantitative, color‐coded images, artificial intelligence	Sensitivity and specificity: 90.16%/80.95% Low‐grade glioma 95.45%/82.14% High grade glioma 90.55%/82.73% Low‐grade as well as high‐grade gliomas
You et al.^29^	In vivo (animals)	1 rat	Cortex, tumors	Quantitative, vascular network map, density map, and density ratio map	Sensitivity 77.7%, specificity 98.2%
Vuong et al.^30^	In vitro (animals)	10 rats	Cerebellar molecular layer, granular layer, white matter, tumors	Quantitative	The light attenuation values for the cerebellar molecular layer, the granular layer, the cerebral white matter, and medulloblastoma were 4.8 ± 0.6 mm^−1^, 7.1 ± 0.3 mm^−1^, 5.2 ± 0.4 mm^−1^, and 7.8 ± 0.4 mm^−1^, respectively.
Chong et al^31^	In vivo (animals)	C57BL/6 mice, 3 cases	Hippocampal tissue white matter	Quantitative	Quantitative comparison of cortical tissue OCT signal attenuation characteristics demonstrated the advantages of 1.7 μm for deep tissue brain imaging, presenting imaging of the hippocampal region and white matter microvascular system in vivo.
Katta et al.^32^	In vivo (animals)	U251 tumor‐bearing nude rats, 5 cases	Tumor vasculature and peritumor vasculature	Qualitative, quantitative, vascular network diagram, vascular reconstruction diagram	Postoperative peritumoral tissue was pathologically verified to show no residual tumor in the tumor cavity
Yecies et al.^33^	In vivo (animals)	U87 tumor‐bearing nude rats	Cortical tracts, white matter tracts, tumors	Qualitative	Modified OCT system with speckle modulation shows tumor finger‐like infiltration borders, white matter fiber bundles, and myelin‐containing axons
Yu et al.^34^	In vitro (human specimen)	18 cases of glioma, 13 cases of meningioma	tumors	Qualitative	Helps to identify tumor type as well as glioma grade
Zhu et al.^35^	In vivo (animals)	U87‐bearing nude, 16 cases	Tumor, peri‐tumor normal tissue	Quantitative	Combined with quantitative autofluorescence technology, the OCT system increases the average sensitivity of the detected specimens from 86.1% to 95.9%
Li et al.^36^	In vitro (animals)	Glioma‐carrying nude rats Normal porcine brain	Tumor‐bearing whole brain	Quantitative	OCT‐guided intelligent robotic arm surgical resection of tumors up to 1.16 mm with 91.7% accuracy
Achkaso et al.^5^	In vitro (human specimen)	215 brain tissue samples	White matter, tumors	Qualitative, quantitative	Alterations in myelinated fibers lead to changes in white matter scattering properties
Yuan et al.^37^	In vivo (animals)	BALB/c nude mice	The mouse brain structures, including cerebral cortex, corpus callosum, caudate putamen, ventral striatum, and thalamus.	Qualitative, quantitative	With OCT high‐resolution imaging capabilities, microneedling offers the opportunity for in vivo assessment of the target lesion and enables precise laser probe placement and optimal in situ ablation planning, potentially leading to better treatment outcomes.
Geng et al.^23^	In vivo (animals)	BALB/c mice a melanoma tumor‐bearing mouse model	The mouse brain and blood vessel, tumors	Qualitative,	P‐INT (a natural fat nanoemulsion) significantly improves the contrast and detection resolution of OCT images and the monitoring of brain diseases and tumors.
Möller et al.^38^	In vitro (human tissue samples)	14 tissue samples	brain metastases and healthy tissue	Qualitative, quantitative	Automatic classification of brain metastases and healthy brain tissue is feasible using OCT imaging, extracted texture features, and machine learning using principal component analysis (PCA) and support vector machines (SVM).

Abbreviations: CNS, central nervous system; OCT, optical coherence tomography; PCA, principal component analysis; P‐INT, a natural fat nanoemulsion; SVM, support vector machines.

Bizheva et al.^26^ first reported an in vitro study on living human brain tissue based on ultrahigh‐resolution optical coherence tomography (UHR OCT) technology, which achieved micrometer‐scale results. In this study, by combining a cutting‐edge Ti:Al_2_O_3_ laser with a free‐space OCT system and utilizing dynamic focusing technology, high‐resolution OCT images in biological tissues and various types of brain tumors, including fibrous meningiomas, migrating meningiomas, and ganglionic gliomas, were obtained. To facilitate histological comparisons and visualization, as well as the identification of morphological features such as microcalcifications (which are typically present only in neuropathological states) and pathological features like enlarged tumor cell nuclei, researchers conducted UHR OCT imaging on multiple types of brain tumors and healthy brain tissues, covering hemorrhagic, fibrous, migrating meningiomas, and ganglionic gliomas.

**Figure 4:**
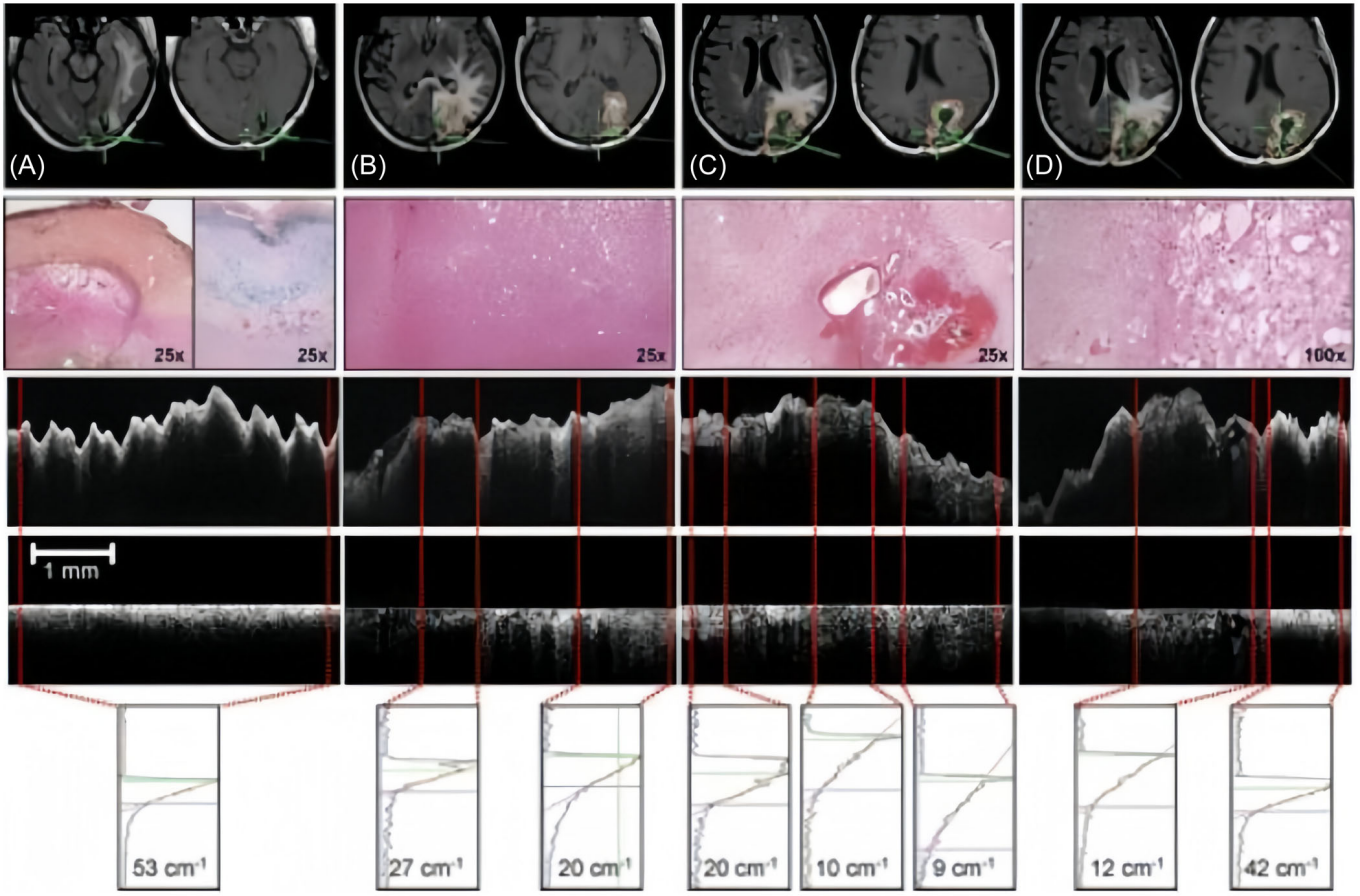
Intraoperative OCT imaging during glioblastoma resection.^7^ (A)Collagen‐rich cortical scar H&E stained (left) with deposition of iron‐pigmented macrophages (right; Berlin blue reaction). (B) Microcystic changes within the tumor infiltration zone. (C) Vascular proliferation and pleomorphic tumor cells. (D) Pleomorphic tumor cells within the tumor center (right) and tumor necrosis (left). H&E, hematoxylin and eosin; OCT, optical coherence tomography. [Color figure can be viewed at wileyonlinelibrary.com]

Bohringer et al.^7^ employed OCT technology to examine 42 intraoperative biopsy specimens from 10 patients, 9 of whom had glioblastoma and 1 with mesenchymal astrocytoma. The OCT system exhibited remarkable capabilities in identifying areas of abnormal light attenuation and lesion microstructures, encompassing microcystic changes, high tumor densities, pleomorphic tumor cell areas, necrotic areas, and collagen fiber‐rich cortical crusting of recurrent tumor tissues. These findings significantly aided in the identification of solid tumors, extensively infiltrated brain tissue, necrotic areas, and normal brain tissue regions (Figure 4). The study suggests that OCT technology holds promise as a surgical assistant in the identification of tumors and the surrounding normal brain tissue. Nevertheless, it is noteworthy that the research did not establish a definitive threshold value for discriminating tumor tissue from peritumoral normal brain tissue.

Kut et al.^27^ used a quantitative OCT technique to analyze 32 fresh human brain tumor specimens (grade II–IV gliomas), pathologically confirming tumor infiltration zone specimens with less than 5% microscopic tumor cells, and 5 fresh normal brain tissue specimens obtained from epileptic patients requiring lobectomy. They quantified light attenuation values in these specimens. At a threshold of 5.5 mm^−1^, the OCT system exhibited remarkable specificity and sensitivity for both high‐ and low‐grade brain cancers. Specifically, for high‐grade gliomas, the specificity reached 100% while the sensitivity stood at 92%. For low‐grade gliomas, the sensitivity peaked at 100% with a specificity of 80%. Diagnostic thresholds tailored specifically for OCT specimen types were formulated, revealing that a threshold of 5.5 mm^−1^ achieved these impressive results. An in vivo validation using a glioma‐bearing rat model (U87 or GBM272 cell line) further confirmed the system's effectiveness, indicating it could facilitate surgeons in achieving over 95% tumor removal, surpassing the 5%–10% residual tumor rate observed in the control group. However, a critical limitation was identified in this study. The distance between the OCT probe and the specimen must be precisely maintained at 0.5 mm during detection, which poses significant challenges in real‐world applications, as it is difficult to maintain a stable 0.5 mm interval between the probe and the specimen's surface. Furthermore, ensuring the detected surgical cavity remains perfectly flat during tumor resection is unlikely, introducing variables that could affect the accuracy of OCT‐based detection and identification.

Vuong et al.^30^ used OCT tomography to probe the whole brain structures of six Ptch+/‐ rats and four control rats. They measured the light attenuation values in regions such as the pathological cerebellar molecular layer, granular layer, cerebral white matter, and medulloblastoma. Based on research results, the light attenuation value for the cerebellar molecular layer was 4.8 ± 0.6 mm^−1^, the granular layer was 7.1 ± 0.3 mm^−1^, and the cerebral white matter was 5.2 ± 0.4 mm^−1^. It is noteworthy that the researchers observed, through OCT imaging, that the attenuation values within the medulloblastoma region (with an average of 7.8 ± 0.4 mm^−1^) were comparable with those of the granular layer, the similarity in light attenuation values observed in medulloblastoma regions and the granular layer may be attributed to the fact that both regions have relatively high cell densities. In contrast, regions with lower to medium cell densities, such as the molecular layer of the cerebellum, demonstrate a lower light attenuation coefficient compared with the former. This suggests a potential positive correlation between cell density and light attenuation coefficient when measuring light attenuation coefficients of different brain tissue regions using OCT.

Almasian et al.^8^ successfully utilized an OCT system to identify tissue types intraoperatively in six patients with gliomas. During the study, they conducted detections on both the cortical and subcortical levels for gliomas and normal brain tissue. The results revealed that the mean attenuation value for normal brain tissue in the cortical layer was 3.8 ± 1.2 mm^−1^, while for gliomas, it was 3.6 ± 1.1 mm^−1^. Similarly, in the subcortical layer, the mean attenuation values were 5.7 ± 2.1 mm^−1^ for normal brain tissue and 4.5 ± 1.6 mm^−1^ for glioma. These findings strongly validate the effectiveness of the OCT system in detecting and distinguishing human brain tumors from normal tissues during neurosurgical operations.

Chong et al.^31^ used FD‐OCT in vivo microscopy with a 1.7 μm supercontinuum light source to noninvasively detect subcortical structures in mice in vivo. A quantitative comparison of OCT signal attenuation characteristics of cortical tissue demonstrated the advantages of the 1.7 μm light source for imaging deep brain tissue, exemplified by clear visualizations of the hippocampal region and the microvascular system in white matter.

Katta et al.^32^ used an OCT angiography system to capture images of gliomas in human glioma‐carrying (U251‐Luc‐RFP) nude mice, obtaining OCT images of tumors before, during, and after electrocoagulation or ablation. The system, when combined with pathological validation, demonstrated the ability to accurately delineate tumor size. Setting a threshold of 5.7 mm^−1^, the system was combined with a fluorescence confocal microscopy system to display angiography, and clearly display tumor‐feeding vessels and peritumoral vasculature. This integration could aid operators in severing tumor blood supply before excision, safeguarding normal brain tissues. Postoperatively pathological analysis confirmed no residual tumor in the tumor cavity.

Yashin et al.^19^ measured and calculated the light attenuation values and forward cross‐scattering values of isolated specimens from 30 patients ex vivo. These specimens encompassed cortex, white matter (intact myelin and damaged myelin), astrocytoma, and glioblastoma (both necrotic and viable). Statistical analysis revealed significant differences between the majority of tumor tissues and white matter and cortex. The study delved into various tissue areas, including tumor zones, tumor‐white matter interfaces, white matter regions, white matter‐cortex boundaries, and cortical regions. The OCT data underwent rigorous analysis and visualization, indicating the promising potential of an orthogonal polarization OCT system for intraoperative differentiation between tumor tissue and white matter tissue.

You et al.^29^ used high‐resolution OCTA and fluorescence images to detect the tumor boundaries in GL261 tumor‐bearing C57BL/6 mice in vivo. By analyzing neovascularization (through OCT angiography), microcirculatory blood flow (through optical Doppler imaging) and tumor proliferative activity (through GFP fluorescence), they found that OCT angiography exhibited superior sensitivity (77.7%) and specificity (98.2%) in identifying tumor neovascularization for accurate tumor boundary determination, compared with the other two modalities.

Yecies et al.^33^ introduced speckle modulation‐optical coherence tomography (SM‐OCT), a cutting‐edge technique that enhances OCT resolution to 10 µm and expands the scanning area. By averaging the mean value of nonspecular areas, the SM‐OCT system eliminates speckle noise, enabling precise identification of cortical layers and white matter fascicles. When applied to the brains of live U87 tumor‐bearing nude mice and BL6 mice, the SM‐OCT system was able to reveal the boundaries of the tumor finger‐like infiltrations, as well as white matter fiber fascicles and myelinated axons.

**Figure 5:**
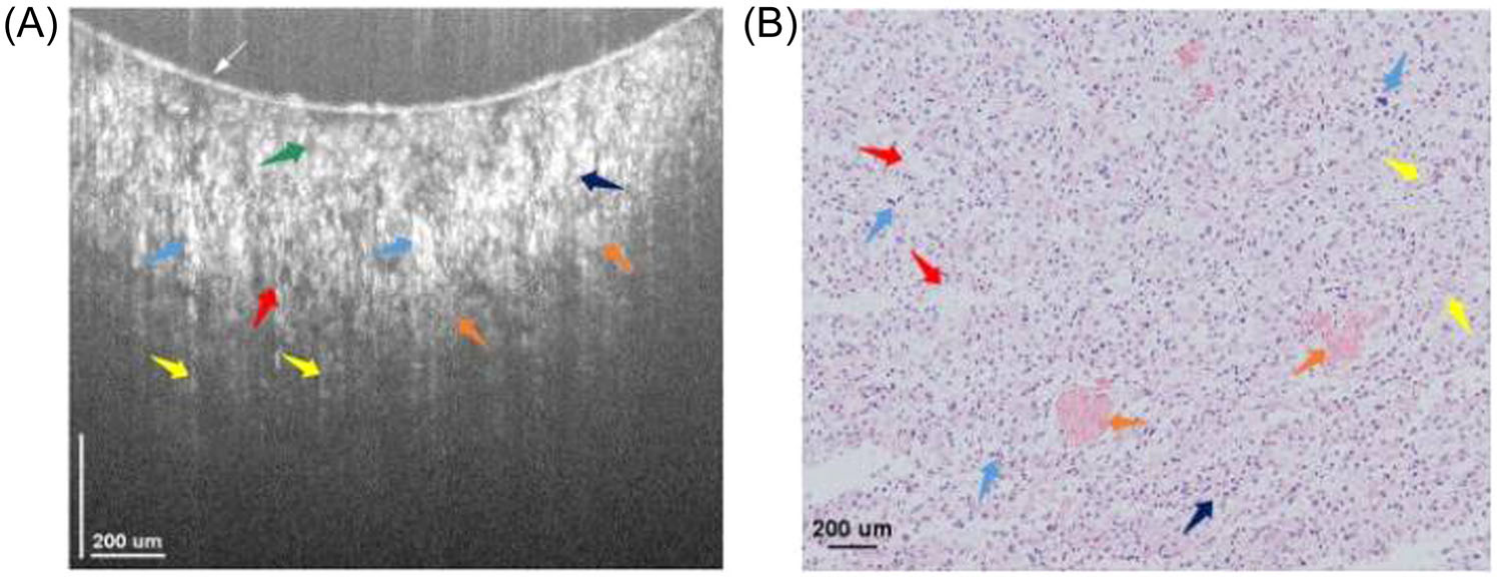
Typical OCT cross‐sectional image of low‐grade glioma tissue.^34^ (A) The image covers a width and depth of 1.5 × 0.7 mm and allows the identification of typical microstructures such as glioblastoma multiforme (blue arrows), mucin‐like stroma (green arrows), and gliomas (orange arrows), as well as vesicles (red arrows) and fibers (yellow arrows). (B) Histology typical histological images of glioma tissue images with hematoxylin and eosin: ×100. OCT, optical coherence tomography. [Color figure can be viewed at wileyonlinelibrary.com]

Yu et al.^34^ employed a micro‐OCT system (μOCT) capable of achieving a high spatial resolution of approximately 2.0 µm to examine 18 fresh glioma samples and 13 fresh meningioma samples. A comparison of the OCT grayscale images with the histological stained with hematoxylin and eosin (HE) revealed that the micro‐OCT system could discern corresponding fibrous structures, whorl formation, and distinct histological features, such as meningeal epithelial tissue, mucin‐like stroma, glioblastoma, glioma, and vesicles. This capability aids in the accurate identification of tumor type and glioma grade (Figure 5).

Zhu et al.^35^ utilized a dual‐modality approach combining quantitative autofluorescence spectroscopy and OCT to detect tumors 16 U87MG tumor‐bearing nude mice in vivo. This system assisted surgeons in differentiating tissue types during surgical procedures. Notably, the combination of quantitative autofluorescence and OCT enhanced the average sensitivity of tumor detection from 86.1% to 95.9% compared with that of the OCT system alone. These findings confirmed that the dual system effectively supports tumor identification and boundary delineation.

Li et al.^36^ pioneered the use of OCT‐guided robotic arm surgery for ex vivo resection of gliomas in nude mice. Their system attained a remarkable precision of 1.16 mm and a commendable tissue classification accuracy of 91.7%. Notably, the error associated with OCT‐guided automated laser ablation was approximately 0.74 mm. Furthermore, the system underwent rigorous testing in a normal porcine brain model, underscoring its potential for accurate and intelligent real‐time diagnosis and treatment.

## 5 THE POTENTIAL OF OCT IN THE DIAGNOSIS AND TREATMENT OF CNS TUMORS

In the diagnosis and treatment of CNS tumors, OCT provides high‐resolution imaging at the micron level, allowing the boundaries between tumor and normal tissue to be clearly identified, providing a more precise and intuitive means of visualizing tumors, which is essential to accurately differentiate between tumor areas and avoid damage to normal tissue. In the treatment of CNS tumors, laser‐based surgical approaches (e.g., using an infrared laser source) allow precise ablation of tumor tissue. Compared with traditional scalpels or surgical forceps, laser surgery allows precise control of the depth and extent of ablation, reduces damage to surrounding normal tissues, and helps reduce postoperative recovery time and complication rates.^39^, ^40^

The combination of OCT technology and laser ablation may play an even greater role in CNS tumor surgery, allowing the surgeon to control the extent and depth of laser irradiation based on the tumor boundaries as shown on the OCT image. The surgeon precisely controls the extent and depth of laser irradiation based on the tumor boundaries as shown on the OCT image. This precise and minimally invasive surgical approach not only improves diagnosis and treatment outcomes but also helps to reduce the recovery time and complication rate after surgery, resulting in a better prognosis and quality of life for patients.

Yuan et al.^37^ reported an ultra‐compact (580 μm OD) therapeutic diagnostic deep brain microneedle that combines OCT imaging with laser ablation, where the imaging function permits real‐time, high‐resolution visualization of tissue microstructure, while the ablation function precisely treats the target tumor tissue. Meanwhile, the microneedle OCT overcomes to some extent the inherent depth limitations of OCT with high axial resolution and better imaging contrast, improving the diagnostic ability of OCT to detect diseased and nondiseased tissues in the deep brain, which could combine with ablative lasers for optimal therapeutic outcomes of deep brain lesions. For future clinical applications, a combination of machine learning algorithms could be considered for real‐time tumor detection and ablation assessment in the deep brain.

## 6 PROSPECTS FOR AI IN OCT SYSTEMS

The OCT scanning could generate a huge amount of data. For instance, for a 5 × 5 × 10 mm^3^ tissue, the number of OCT B‐scan grayscale images could reach 400–500, with a corresponding pixel count reaching 8^10^7^. Manual analyses are tasking, difficult, cumbersome, and time‐consuming. AI is suitable for processing large datasets, increasing efficiency, and providing timely analysis, making them particularly useful for distinguishing between different tissue types, such as tumor and nontumor tissues, in OCT images. In a study by Juarez‐Chambi et al.,^28^ the sensitivity of the in vitro in situ detection of human glioma infiltration using an AI‐assisted OCT system exceeded 90%, indicating that the system was able to accurately identify the vast majority of glioma infiltration. Using machine learning feature recognition techniques on a single line (A‐line) of an OCT vertical scan, this approach provides an effective tool for the automated analysis of medical images. Additionally, the study employed a larger data set, comprising tumor cavity tissues from 21 patients. Of these, 194 OCT datasets were utilized for training, while 295 were employed for validation, which facilitated the refinement of the AI algorithm. However, it is flawed in that (1) The performance of the machine learning feature recognition algorithm in distinguishing high‐grade gliomas, low‐grade gliomas, and nontumorous regions had a sensitivity of 90.55% and a specificity of 82.73%, without mentioning the accuracy rate, which is crucial for evaluating models in the field of medical diagnosis, where the false positive rate must be strictly controlled. Therefore, even if the model has a high sensitivity and specificity, the possibility of a low precision rate cannot be ruled out. In other words, although the model may perform well in distinguishing between diseased and nondiseased individuals, it may have a high false positive rate when predicting specific disease categories (e.g., high‐grade glioma or low‐grade glioma).^41^ (2) The methodology employed to remove the gray‐scale map displayed above the specimen failed to consider the crucial parameter of the distance between the probe and the specimen, which is therefore excluded from subsequent calculations. Previous studies and literature have demonstrated that the distance between the probe and the specimen surface has a significant impact on the gray‐scale map data, including the absolute size of the gray‐scale value, the attenuation value, and other indexes. Excluding this parameter may result in reduced accuracy, sensitivity, and specificity of the AI algorithm, especially after preprocessing when the top layer is excluded from calculations.^42^

**Figure 6:**
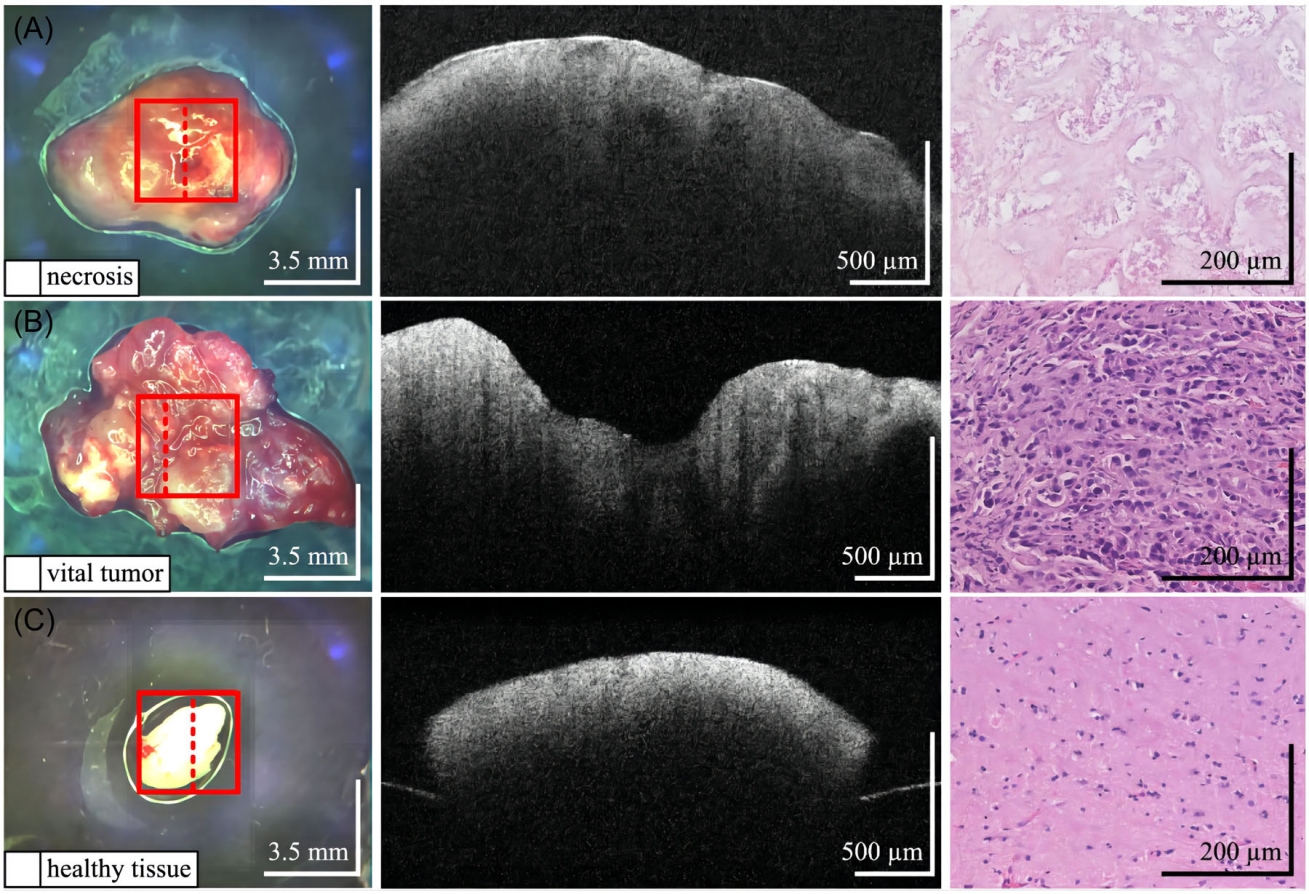
The machine learning‐based tissue types differentiation task by OCT system, including necrosis, significant tumor, and healthy tissue.^38^ Examples of analyzed tissue types: (A) Necrosis, (B) Vital tumor, and (C) Healthy tissue. The left panel represents a general photograph of the tissue sample, with the square in the image marking the mid‐column OCT B‐scan on the surface of the acquired image of the entire volume; the dotted line indicates the actual location of the OCT B‐scan shown. Photos show tissue samples with OCT B‐scans (middle panel) and histology slides (right panel). OCT reveals smooth, healthy tissue (C) versus irregular necrosis (A) and tumor (B). Histology shows acellular necrosis (A), high cellularity in tumor (B), and structured healthy tissue (C) with glial cells. Dashed lines in photos mark OCT scan positions. Automated tissue classification was verified by histological examination to accurately classify necrotic, vital tumor, and healthy brain tissue. OCT, optical coherence tomography. [Color figure can be viewed at wileyonlinelibrary.com]

Möller et al.^38^ distinguished brain cancer metastases from healthy brain tissue in an ex vivo setting by applying texture analysis and machine learning algorithms to OCT images. The strength of this approach lies in its ability to utilize a wide range of texture parameters, employing both structural and statistical features, which can effectively distinguish tissue types based on differing image patterns (Figure 6). The results of the study distinguished significant tumors from healthy brain tissue with 95.75% accuracy, suggesting that the application of AI to OCT systems has developmental advantages for optimizing the extent of tumor resection and minimizing the risk of local recurrence. However, the disadvantage is that this approach requires a large number of samples to support it, and uses rather complex texture analysis and machine learning algorithms for automatic classification.

First of all, a large number of samples are imperative to support machine learning algorithms for the application of AI in OCT systems. OCT is a highly sophisticated imaging technique, and each piece of tissue, although hundreds of images can be generated during scanning, cannot be effectively expanded in terms of sample size as these images are all extremely similar. To address this, new approaches, such as advanced feature extraction and recognition methods, are necessary to enable training and testing with a limited sample size. For example, our unpublished data revealed that analyzing the OCT grayscale data and extracting specific features based on the A‐line and its trend lines, light attenuation values, and the grid method can improve classification accuracy. Using the XGBoost algorithm, we were able to classify gliomas and peritumoral nontumorous brain tissues, with a high precision rate of 94.0%, specificity rate of 77.07%, and sensitivity rate of 99.06% after analyzing glioma specimens from only 17 patients.

Additionally, there may be a drastic increase in the difficulty of machine learning to achieve the identification of tissue types in cases where both tumor and peri‐tumor tissue are present within the same grayscale image as the prerequisite for achieving this goal relies on effective tissue labeling, and also in cases where there are special cases of edema, hemorrhage, coagulation scarring and other special circumstances of the tissue type, which are difficult to be identified by the AI technique. In the future, there is a critical need for research focusing on new AI methods capable of identifying tumor tissues in OCT gray‐scale maps with improved sensitivity, specificity, and accuracy. Meanwhile, more studies are warranted to explore the OCT grayscale imaging features of brain tissues that have edema, hemorrhage, or coagulation scarring to ensure the smooth utilization of AI in the OCT system.

## 7 CONCLUSIONS AND PROSPECTS

**Figure 7:**
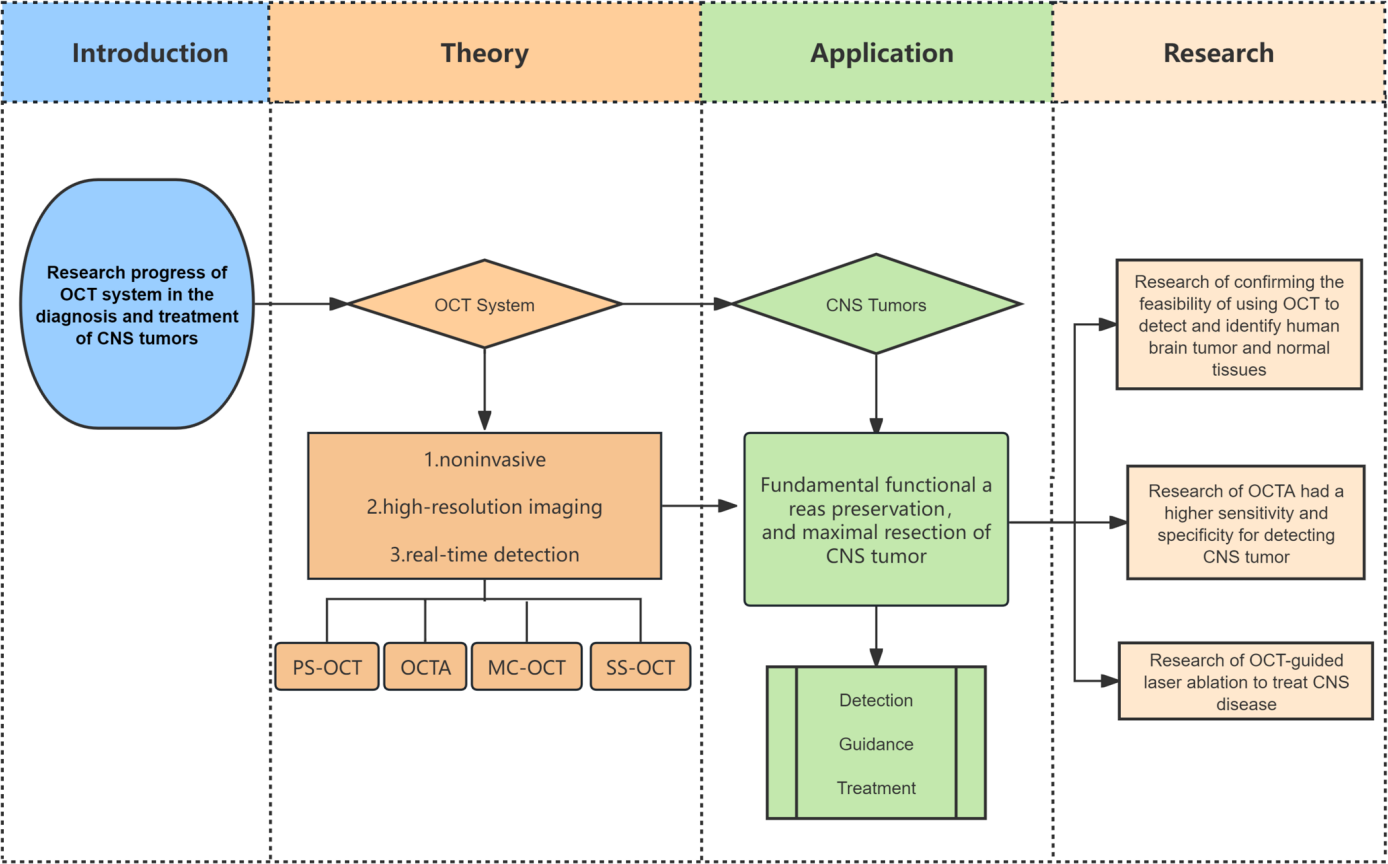
Mind map of the full text. With the advantages of high‐resolution imaging, efficient image acquisition, intraoperative real‐time detection, and radiation‐free and noninvasive characteristics, OCT provides accurate diagnosis and effective intraoperative guidance for the minimally invasive diagnosis and treatment of CNS tumors. These methods include swept‐source OCT, polarization‐sensitive OCT, orthogonal polarization OCT, Doppler OCT, and OCT angiography. This review summarizes the relevant literature and the research progress on the roles and applications of OCT in CNS tumors, provides a synopsis of the advantages of the OCT system in the surgical guidance and diagnosis of CNS tumors based on the detection of the OCT system, and introduces relevant clinical studies on the integration of the OCT system with other neurosurgical therapeutic techniques for the treatment of CNS tumors to provide prospects for future research directions. CNS, central nervous system; OCT, optical coherence tomography. [Color figure can be viewed at wileyonlinelibrary.com]

The OCT system, owing to its distinctive characteristics, offers numerous advantages over conventional methods for detecting tumor boundaries. These advantages stem from its noncontact, noninvasive nature, high‐resolution imaging, real‐time display capabilities, robust continuity, portability, and the elimination of the need for contrast agents. Notably, OCT technology has demonstrated significant potential in detecting nervous system tumor boundaries, guiding neurosurgical procedures, and enabling minimally invasive diagnosis and treatment, particularly when integrated with laser ablation (Figure 7).

Enhancing the performance of OCT systems, particularly in terms of detection depth, will pave the way for expanded applications in detecting neurological tumor boundaries. The development of integrated systems that leverage multiple OCT techniques, coupled with conventional imaging modalities like MRI and intraoperative navigation techniques, promises to further broaden the real‐time detectability of OCT technology. This represents a new frontier in the utilization of OCT for intracranial tumor resection.^43^, ^44^

The combination of OCT and laser ablation represents an innovative strategy for the diagnosis and management of brain tumors. This minimally invasive technique not only facilitates precise and intelligent resection of CNS tumors but also enables a wide range of associated therapeutic interventions. Furthermore, the incorporation of AI techniques into OCT systems holds immense value for advancing research and development in this field.^45^

The future trajectory of OCT research will be profoundly shaped by advancements in computing power and AI. Graphical processing units, for instance, offer promising prospects for high‐speed numerical processing with compact, energy‐efficient, and cost‐effective hardware solutions.^46^ The combination of OCT detection and diagnostic techniques with robotic surgical treatment of intracranial tumors has been developed based on AI.^47^ These are considered to be the future research directions of OCT. What is more, applying machine learning to OCT imaging can prospectively provide surgeons with more information about the tumor tissue to optimize the extent of tumor resection, with better preservation of fundamental brain areas and may minimize the risk of local recurrence.

## AUTHOR CONTRIBUTIONS

Jiuhong Li was responsible for conceptualizing the research, writing the original draft, and proposing and structuring the overall research goals and objectives. Ziba Ayi contributed to writing the original draft and was responsible for its compilation and revision. Gonggong Lu contributed to writing the original draft, prepared, and analyzed the study data. Haibo Rao provided resources, including analytical tools and relevant data. Feilong Yang acquired funding and provided financial support for the project leading to this publication. Jing Li participated in project management, overseeing the planning and execution of research activities, as well as coordination and management responsibilities. Jiachen Sun conducted formal analysis and analyzed the study data. Junlin Lu contributed to writing and revising the review. Xulin Hu was responsible for the leadership and supervision of the literature reviewing, as well as the visualization of the results. Si Zhang contributed to writing revisions and shared management and coordination responsibilities for the planning and execution of research activities. Xuhui Hui participated in project management, and was responsible for overseeing the planning and execution of research activities, as well as management and coordination responsibilities.

## CONFLICT OF INTEREST STATEMENT

Jing Li, who is affiliated with Chengdu Incrpeak Optoelectronics Technology Co., Ltd., Optoelectric Industrial Park, Chengdu 610207, China, declares as only being the co‐author, and no such hidden identity/information/consequence which will become the influence of this study. The remaining authors declare no conflict of interest.

## ETHICS STATEMENT

Ethics approval was granted by the Institutional Animal Care and Use Committee of West China Hospital of Sichuan University (20230310031). Figure 3 inserted in the article shows experimental data was from our research team.

## Data Availability

Data sharing is not applicable to this article, as no new data were created or analyzed in this study.
